# Surgery, Non-Surgical Dilatation for Bile Duct Strictures

**DOI:** 10.1155/1991/69876

**Published:** 1991

**Authors:** P. J. Kestens, J. F. Gigot

**Affiliations:** Digestive Surgery Department Medical School Universite Catholique de Louvain Cliniques Universitaires Saint-Luc Avenue Hippocrate 10 Bruxelles B-1200 Belgium


*HPB Surgery*, 1991, Vol. 3, pp. 139–144
Reprints available directly from the publisher
Photocopying permitted by license only

© 1991 Harwood Academic Publishers GmbH
Printed in the United Kingdom

### HPB INTERNATIONAL

EDITORIAL & ABSTRACTING SERVICE JOHN TERBLANCHE, EDITOR
Department of Surgery,
Medical School · Observatory 7925
Cape Town · South Africa
Telephone: (021) 47-1250 Ext. 229
Telefax (021) 47-8955

### SURGERY, NON-SURGICAL DILATATION FOR BILE DUCT STRICTURES

### ABSTRACT

*Pitt HA, Kaufman SL, Coleman J, White RI, Cameron JL (1989). Benign Postoperative Biliary Strictures. Annals of Surgery; 210:417–425.*

**At The Johns Hopkins Hospital from 1979 through 1987, 42 patients had 45 procedures for benign postoperative biliary strictures. Three patients were managed with both surgery and balloon dilatation. Twenty-five patients underwent surgical repair with Roux-Y choledocho- or hepaticojejunostomy with postoperative transhepatic stenting for a mean of 13.8 ± 1.3 months. Twenty patients had balloon dilatation a mean of 3.9 times and were stented transhepatically for a mean of 13.3 ± 2.0 months. The two groups were similar with respect to multiple parameters that might have influenced outcome. Mean length of follow-up was 57 ± 7 and 59 ± 6 months for surgery and balloon dilatation, respectively. No patients died after any of the procedures. The same definition of a successful outcome was applied to both groups and was achieved in 88% of the surgical and in only 55% of the balloon dilatation patients ($p < 0.02$). Significant hemobilia occurred more often with balloon dilatation (20% *vs* 4%, $p < 0.02$). The total hospital stay and cost of balloon dilatation was not significantly different from surgery. We conclude that surgical repair of benign postoperative strictures results in fewer problems that require further therapy. Nevertheless balloon dilatation is an alternative for patients who are at high risk or who are unwilling to undergo another operation.**

### PAPER DISCUSSION

KEYWORDS: Bile duct stricture, biliary tract stricture, radiological dilatation, hepaticojejunostomy

To our knowledge, this article is the only one in the literature, comparing the surgical and non surgical treatment of benign postoperative strictures of the bile ducts. Indeed, at admission, the patients are managed by a team of surgeons and radiologists who decide how to treat them: unfortunately, this was not done on a randomized basis and the authors don't mention why a particular patient is treated by surgery or not. Nevertheless, the treatment, either surgical or non surgical, was not decided according to the operative risk as it is in most published papers. On the contrary, there is no significant difference between the two series with regard to the presence of intra-hepatic lithiasis, associated cirrhosis, portal hypertension, presence of biliary fistulae or number of previous operative procedures.

However, one can observe that the "medical" group contains more type I and II strictures than the "surgical" one so that surgery has more type III patients to treat who represent a more difficult problem with a poorer prognosis.

This last observation enhances of course the superiority of the surgical treatment which produces less recurrences at the same cost.

We would like to make a few more comments:

1. It is surprising that in the dilatation group, the authors put the accent on the dilatation more than on the stenting. This stenting is left in place for quite a long period (1,3 months). May we suggest to use other types of stents such as expanding wall stents or gianturco stents for which no long term results have been published yet, but their principle is ingenious.

2. There are some questions about the surgical technique which is not given in detail. It is not clearly stated that the stricture was completely resected and the anastomosis performed above the lesion on a "healthy" bile duct. In type III and IV, one needs to dissect the right and left main bile duct high in the hilum, the anastomosis being performed on these ducts. Sometimes, one has to restore a previously damaged junction by anastomosing the right and left duct together to a Roux-en-Y loop or by making a double bilio-digestive anastomosis. HEPP[1] and BISMUTH[2] in France, have described this technique in detail and insist on the necessity to approximate biliary mucosa: to digestive mucosa this seems to be the basic principle to avoid restenosis. CAMERON and co-workers[3], on the contrary, find that "in most instances a mucosa-to-mucosa anastomosis cannot be fashioned".

3. Stenting of the anastomosis is performed as a rule for a long period ($>$ 1 year). We feel that this manoeuver is not at all mandatory when a wide ($>$ 1 cm) mucosa-to-mucosa anastomosis is performed.

Moreover, the chronic irritation by the stent can be harmful and provoke a secondary stenosis.

4. The follow-up period (5 years) is reasonably long but not long enough to yield definitive results: our group has observed that a recurrence of the stricture can occur even after 10 years.

In conclusion, this paper is an attempt to compare two ways of treatment, surgical or non surgical. More of these objective, well documented and honest reports should be produced in hepato-biliary and pancreatic diseases in order to show that surgery is still the best treatment in many instances in terms of quality of the results, cost and rapid rehabilitation compared to non surgical methods such as interventional radiology or "operative" endoscopy.

P.J. Kestens and J.F. Gigot
Digestive Surgery Department
Medical School Universite Catholique de Louvain
Cliniques Universitaires Saint-Luc
Avenue Hippocrate 10
B–1200 Bruxelles, Belgium

*References*

1. Bismuth, H. and Lazorthes, F. (1981) Les traumatismes operatoires de la voie biliaire principale. Monographie de l'Association Française de Chirurgie (AFC). Masson
2. Hepp, J. (1985) Hepaticojejunostomy using the left biliary trunk for iatrogenic biliary lesions: the French connection. *World J. Surg.* **9**, 507–511
3. Cameron, J.L., Gayler, B.W. and Zuiedma, G.D. (1978) The use of silastic trans-hepatic stents in benign and malignant biliary strictures. *Ann. Surg.* **188**, 552–561

### PERIHEPATIC PACKING IN THE MANAGEMENT OF LIVER TRAUMA

### ABSTRACT

*Hollands, M.J., Little, J.M. (1989) Perihepatic packing: Its role in the management of liver trauma. Aust NZ Surg 59: 21-24.*

**Perihepatic packing was used in 25 of 197 (12.7%) patients presenting with liver trauma to Westmead Hospital over an 8 year period. Packing was used either to provide temporary haemostasis prior to transfer or as part of a definitive treatment plant at this hospital. Thirteen patients were packed prior to transfer. Only two were unstable on arrival, one of whom died. They were compared with 18 'comparison' patients with liver injuries of similar severity. In this group 10 were unstable on arrival ($P=0.027$), nine of whom died ($P=0.015$). Packing was used as part of a definitive treatment plan at Westmead on 17 occasions. Four patients were coagulopathic and five had also been packed prior to arrival. Eight of this group died.**

**Packing is a convenient and safe way of controlling major hepatic haemorrhage prior to transfer to a tertiary referral centre. It may also be part of a definitive treatment plan to control hepatic bleeding especially as many patients arrive with a coagulopathy or develop a coagulopathy during the course of surgery to control bleeding. Packing will control haemorrhage until the coagulopathy has been corrected.**

